# Supplementary material for: Liver Transplantation for HCC in HIV-Infected Patients: Long-Term Single-Center Experience
Source: Cancers (Basel). 2021 Sep 21;13(18):4727. doi: 10.3390/cancers13184727 (PMC8471924; doi:10.3390/cancers13184727)
Supplement: Supplementary file 1 [file cancers-13-04727-s001.zip › cancers-1360798-supplementary/propensity score file2.pdf]

Table S1. Study population after propensity score matching (PSM).

POPULATION STUDY = 61						
Variable		HIV group n = 32		Control Group n = 29		p Value
		number	%	number	%	
SEX	M	29	90.6	24	82.8	ns
	F	3	9.4	5	17.2	
AGE	mean & sd	50.3	± 5.676	53.3	± 9.4	ns
CHILD	A /B					ns
	C	14		11		
MELD at LT	mean & sd	17.6	± 9.6	15.2	± 5.9	ns
BMI	mean & sd	23.9	± 3.7	26.3	± 3.07	ns
TYPE OF LT	Isolate	31	96.9	29	100	ns
	Com-bined Kidney-Liver	1	3.1	0		
ETIOLOGY	HCV	22	68.7	22	75.9	ns
	HBV	10	31.3	7	24.1	ns
	Alcohol	0		0		ns
	Biliary	0		0		ns
	Metabolic	0		0		ns
	Other	0		0		ns
WAITING LIST TIME days	mean & sd	234	± 150	158.6	±112	ns
RE-OLT	YES	2	6.2	3	10.3	ns

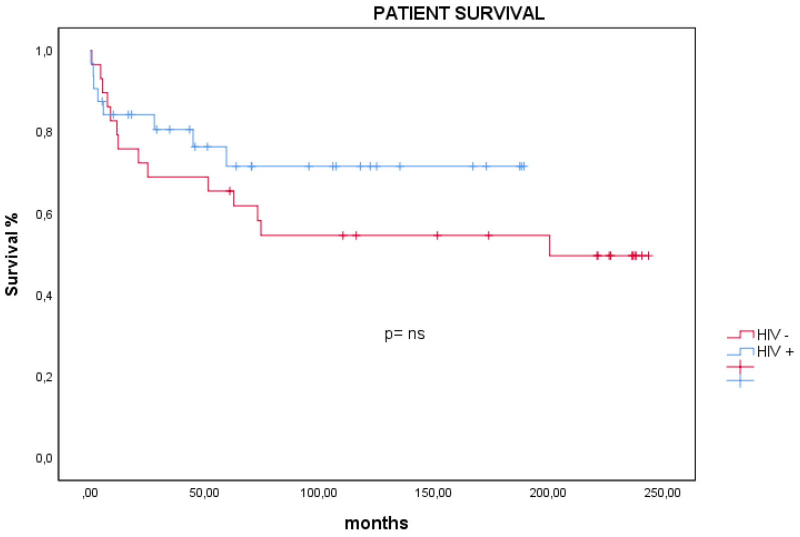

Figure S1. Overall survival in HIV+ and HIV − group.

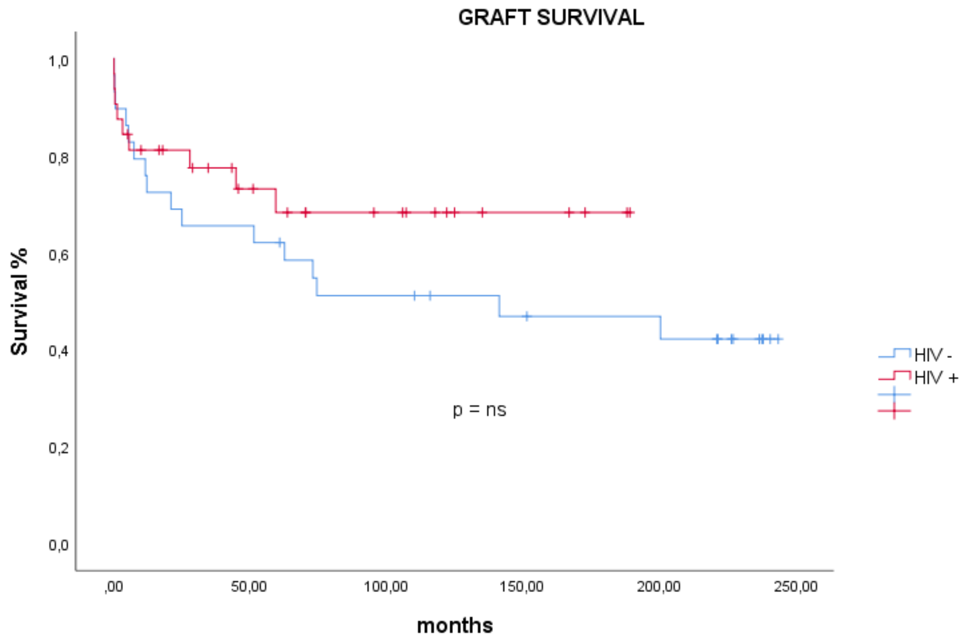

Figure S2. Graft survival in HIV + and HIV − group.

Table S2. Univariate e multivariate analysis of factor impacting the patient survival post-LT in the population

**Variable**	**Scote**	**gl**	***p* value**
HIV +	2.261	1	0.133
AGE OF RECIPIENTS	0.547	1	0.460
SEX	0.894	1	0.344
HCV	2.773	1	0.096
HBV	3.322	1	0.068
WAITING LIST TIME days	0.184	1	0.668
MELD	4.579	1	0.032
TOTAL ISCHEMIA TIME	0.133	1	0.715
DONOR AGE	1.318	1	0.251
RE-oltx	0.558	1	0.455

Cox regression analysis

Variable	B	SE	Wald	gl	*p* value	HR	95.0% CI for HR	
							Min	max
HIV +	−1.282	0.631	4.133	1	0.042	0.278	0.081	0.955
MELD	0.091	0.035	6.672	1	0.010	1.096	1.022	1.174
