# Supplementary material for: Liver Transplantation for HCC in HIV-Infected Patients: Long-Term Single-Center Experience
Source: Cancers (Basel). 2021 Sep 21;13(18):4727. doi: 10.3390/cancers13184727 (PMC8471924; doi:10.3390/cancers13184727)
Supplement: Supplementary file 1 [file cancers-13-04727-s001.zip › cancers-1360798-supplementary/univariate analysis file1.pdf]

Table S3. Variables enter in Cox regression for Overall survival pos LT

	Score	gl	*p* value
Waiting list time days	0.894	1	0.344
MELD	1.295	1	0.255
HIV status	0.912	1	0.340
HCV	5.289	1	0.021
HBV	2.866	1	0.090
Type of LT	0.540	1	0.462
Donor age	0.654	1	0.419
Total ischemia time	1.997	1	0.158
RE-oltx	1.087	1	0.297
mTOR drugs	.	0	.
SEx	3.420	1	0.064
Age of recipient	0.380	1	0.538

Table S4. Variables enter in Logistic regression for HCC recurrence post LT

		score	gl	*p* value
Variables	HIV status	0.101	1	0.751
	HCC grading (G3-G4)	11.146	1	0.001
	Age recipient	0.148	1	0.700
	HCV	0.160	1	0.689
	HBV	1.083	1	0.298
	AFP	0.055	1	0.815
	HCC Number	17.460	1	0.000
	HCC Diameter (largest nodule)	15.248	1	0.000
	San Francisco criteria	16.726	1	0.000
	Microvascular invasion	25.399	1	0.000
